# Hitting the Wall in Marathon Running: An Integrative Psychophysiological and Durability-Based Review

**DOI:** 10.3390/jfmk11030291

**Published:** 2026-07-26

**Authors:** Gerasimos V. Grivas, Walaa Jumah Alkasasbeh, Adam Tawfiq Amawi

**Affiliations:** 1 Physical Education and Sports, Division of Humanities and Political Sciences, Hellenic Naval Academy, 18539 Piraeus, Greece; 2 Department of Physical Education, School of Sports Sciences, The University of Jordan, Amman 11942, Jordan; walaakasasbeh1991@yahoo.com; 3 Department of Movement Sciences and Sports Training, School of Sports Science, The University of Jordan, Amman 11942, Jordan; a.amawi@ju.edu.jo

**Keywords:** durability, hitting the wall, marathon running, neuromuscular fatigue, pacing, perceived exertion, psychophysiology, thermoregulation

## Abstract

**Background/Objectives**: Hitting the wall (HTW) is a common and performance-limiting phenomenon in marathon running, traditionally attributed to glycogen depletion and reduced carbohydrate availability. However, the persistence of late-race performance collapse despite advances in fueling strategies suggests that HTW may not be explained by a single metabolic mechanism. Instead, HTW may represent a psychophysiological performance-collapse phenomenon arising from the interaction between physiological strain, perceived exertion, pacing regulation, and the athlete’s capacity to preserve function under prolonged stress. **Methods**: This narrative review aimed to synthesize the physiological and psychophysiological mechanisms contributing to HTW and to propose an integrative durability-based framework for understanding late-race performance deterioration during marathon running. **Results**: Current evidence indicates that HTW may emerge from the cumulative interaction of metabolic stress, thermoregulatory strain, dehydration, gastrointestinal dysfunction, neuromuscular fatigue, biomechanical deterioration, perceptual responses, and pacing errors. These stressors may progressively increase perceived effort, impair pace regulation, and reduce the athlete’s capacity to preserve physiological function and sustain the intended race pace. Within this framework, durability represents the ability to resist deterioration in physiological characteristics and performance capacity during prolonged exercise and may help explain why runners with similar fitness levels experience different late-race outcomes. **Conclusions**: HTW should therefore not be viewed simply as running out of fuel. Rather, it may represent the manifestation of reduced durability and psychophysiological dysregulation arising from multiple interacting stressors during marathon running. Strategies aimed at improving durability through marathon-specific training, optimized fueling, gastrointestinal tolerance, thermoregulatory preparation, neuromuscular resilience, pacing regulation, perceptual awareness, and individualized monitoring may reduce susceptibility to HTW.

## 1. Introduction

Marathon running represents one of the most demanding endurance events, requiring athletes to sustain a high percentage of their aerobic capacity for approximately two to several hours while coping with progressive metabolic, thermoregulatory, neuromuscular, gastrointestinal, perceptual, and behavioral stress [1,2,3]. Although substantial advances have been made in training methods, nutritional strategies, footwear technology, and performance monitoring, many runners continue to experience a sudden and pronounced decline in performance during the latter stages of the race, a phenomenon commonly referred to as “hitting the wall” (HTW) [4]. From a psychophysiological perspective, HTW may be viewed as a late-race performance-collapse phenomenon in which physiological strain, perceived exertion, and pacing regulation interact to impair the athlete’s ability to sustain the intended race pace [5,6,7].

Traditionally, HTW has been attributed primarily to glycogen depletion [8,9]. Early physiological models proposed that reductions in muscle and liver glycogen availability limit carbohydrate (CHO) availability, increase reliance on fat oxidation, elevate perceived exertion, and reduce the ability to sustain marathon pace [8]. Although fat stores are abundant, fat oxidation provides ATP at a slower rate than CHO metabolism; therefore, a greater reliance on fat as exercise progresses may limit the rapid energy turnover required to maintain marathon race pace [8]. Consequently, CHO loading before competition and CHO ingestion during exercise became central components of marathon preparation and race-day nutrition strategies [10]. More recently, contemporary endurance nutrition has emphasized higher CHO intake rates in selected well-trained athletes, with intakes approaching 120 g·h^−1^ potentially tolerated after appropriate gut training [11,12,13,14,15]. However, both performance benefits and gastrointestinal tolerance remain highly individualized. Nevertheless, late-race performance collapse continues to occur despite improved fueling practices.

The persistence of HTW despite advances in nutrition suggests that glycogen depletion alone cannot fully explain the phenomenon. Large-scale analyses of marathon pacing demonstrate that substantial late-race slowing is common, yet the magnitude of performance decline varies considerably among runners [4]. This variability points toward additional mechanisms beyond substrate availability. In particular, the concept of durability has gained attention as a determinant of endurance performance, referring to the ability to resist deterioration in physiological characteristics and performance capacity during prolonged exercise [16]. Emerging evidence indicates that physiological thresholds, running economy (RE), heart-rate dynamics, respiratory responses, and neuromuscular function may deteriorate during prolonged exercise and contribute to performance decline beyond traditional measures obtained under rested conditions [16,17,18,19]. These physiological changes may also alter perceived effort and pace regulation, thereby linking durability to the psychophysiological control of marathon performance [5,20].

Other physiological systems may also contribute to HTW. Thermoregulatory strain, dehydration, cardiovascular drift, and elevated core temperatures can impair exercise capacity, increase perceived exertion, and alter pacing behavior, especially in warm or humid environments [21,22,23,24]. Neuromuscular fatigue and biomechanical deterioration may reduce force production, impair movement efficiency, and increase the energetic and perceptual cost of running [25,26,27,28]. Gastrointestinal dysfunction may compromise CHO and fluid intake, reduce nutrient tolerance, increase discomfort, and interact with heat stress, dehydration, and high-CHO fueling strategies during prolonged endurance exercise [29,30,31]. These mechanisms may not only impair physiological function but also influence behavioral regulation by increasing the perceived difficulty of maintaining pace [5,32].

Collectively, these findings suggest that HTW should no longer be viewed solely as the consequence of glycogen depletion. This review proposes that HTW represents an integrative psychophysiological failure of durability, whereby the cumulative interaction of metabolic, thermoregulatory, neuromuscular, gastrointestinal, hydration-related, perceptual, and pacing-regulatory stressors exceeds the athlete’s capacity to sustain the intended marathon pace. Therefore, the purpose of this narrative review is to synthesize the physiological and psychophysiological mechanisms underlying HTW and to propose an integrative durability-based framework for understanding late-race performance collapse. By synthesizing current evidence, this review also aims to identify practical strategies that may reduce susceptibility to HTW during marathon running.

## 2. Defining the Marathon Wall

### 2.1. Historical and Operational Perspectives

The phenomenon commonly known as HTW has long been recognized as one of the defining challenges of marathon running [4,33]. Although the term is widely used among runners, coaches, and researchers, its scientific interpretation has evolved over time [4,33,34]. Historically, HTW was viewed primarily as a metabolic event resulting from progressive CHO depletion during prolonged exercise. Early physiological models proposed that reductions in muscle and liver glycogen availability limit the capacity to sustain marathon pace, leading to marked slowing during the latter stages of the race [8,10].

Several colloquial expressions have emerged to describe this phenomenon, including “bonking”, “blowing up”, and “running out of gas” [4]. Although these terms are often used interchangeably, they may reflect different interpretations of late-race collapse. Bonking is traditionally associated with severe glycogen depletion and low glucose availability [8,10], whereas blowing up is more commonly used to describe performance collapse following an unsustainably aggressive pacing strategy [4]. Despite these distinctions, all of these terms describe a substantial reduction in the athlete’s ability to maintain the desired exercise intensity during the latter stages of endurance competition [4,33]. From a psychophysiological perspective, these descriptions also reflect the interaction between physiological strain, perceived effort, and behavioral regulation during prolonged exercise [5,32,35].

Because HTW includes both subjective and objective components, its operational definition remains challenging. Early studies relied largely on runners’ self-reported experiences and highlighted features such as generalized fatigue, unintentional slowing, heavy legs, reduced concentration, desire to walk, and a shift in attention toward simply finishing the race [33,34]. In this context, “heavy legs” refers to the subjective perception that limb movement has become mechanically difficult, poorly responsive, or unusually effortful, rather than to a single isolated mechanism [33,34]. These subjective features are important because endurance performance is regulated not only by physiological capacity, but also by the athlete’s perception of effort and willingness or ability to continue sustaining a given pace under increasing strain [5,35]. More recently, Smyth [4] used large-scale marathon split data to identify runners who experienced substantial and sustained reductions in running speed during the latter stages of the race. This approach allows HTW to be quantified across large populations, but pacing-based definitions also have limitations. Late-race slowing may reflect injury, tactical decisions, environmental conditions, course elevation, or loss of motivation, whereas some athletes may experience severe physiological or psychophysiological distress without meeting a predefined pacing threshold [4,16].

For the purposes of this review, HTW is defined as a sudden and disproportionate decline in running performance during the latter stages of the marathon, characterized by an inability to maintain the intended pace and reflecting the cumulative interaction of physiological, perceptual, and behavioral stressors. This definition incorporates three key elements: (i) an objective performance component, expressed as abrupt or sustained pace reduction; (ii) a subjective component, including severe fatigue, increased perceived effort, reduced concentration, and reduced capacity to continue at the intended speed; and (iii) a mechanistic component, recognizing that HTW may arise from metabolic, thermoregulatory, neuromuscular, gastrointestinal, psychological, and pacing-related factors [4,8,16,21,31]. Accordingly, HTW may be best conceptualized as a psychophysiological performance-collapse phenomenon in which increasing physiological strain is translated into higher perceived effort, impaired pace regulation, and a reduced capacity to sustain the chosen marathon speed [5,32,35].

### 2.2. The Marathon Wall Versus Normal Fatigue

Fatigue is an expected consequence of marathon running and develops progressively as race duration increases. Most runners experience gradual physiological and perceptual changes, including glycogen utilization, cardiovascular drift, thermoregulatory strain, neuromuscular fatigue, and increases in rating of perceived exertion (RPE) [16,25]. Therefore, some reduction in pace during the latter stages of the marathon may reflect normal fatigue rather than HTW.

A critical distinction must therefore be made between normal marathon fatigue and HTW. Normal fatigue is typically characterized by a gradual and relatively predictable increase in physiological strain, perceived effort, and, in some cases, a modest decline in running speed. In contrast, HTW is associated with a disproportionate and functionally meaningful deterioration in performance that exceeds what would normally be expected from race duration alone [4,33]. Athletes often describe HTW as a sudden inability to sustain the intended pace despite continued effort and motivation, accompanied by extreme exhaustion, heavy legs, impaired concentration, and a strong desire to slow down or walk [33,34]. The sensation of “heavy legs” may reflect the combined effects of impaired muscle perfusion, metabolite accumulation, reduced force-generating capacity, and impaired neuromuscular control. In particular, acidosis-related reductions in muscle pH may interfere with calcium–troponin interactions, reduce myofibrillar calcium sensitivity, impair excitation–contraction coupling, and make efficient muscle contraction more difficult [36,37]. These symptoms suggest that HTW involves not only peripheral physiological fatigue, but also a marked disruption in the psychophysiological regulation of effort and pace [5,32,35].

From a pacing perspective, not every positive split should be interpreted as evidence of HTW. Small reductions in running speed may reflect appropriate physiological regulation, environmental conditions, course characteristics, or tactical pacing decisions. In contrast, HTW is more commonly associated with substantial and sustained late-race slowing that significantly compromises performance [4]. This distinction is particularly important when interpreting marathon split data, as some degree of performance decline is commonly observed even among highly trained runners. Thus, the classification of HTW should consider both the magnitude of performance decline and the accompanying perceptual and behavioral features, including excessive perceived effort, impaired concentration, and reduced ability to maintain the intended pace.

Accordingly, HTW should be viewed not simply as an extension of normal marathon fatigue, but as a distinct psychophysiological performance-collapse phenomenon characterized by a marked reduction in the athlete’s ability to sustain marathon pace. This distinction provides the foundation for examining the physiological, perceptual, and behavioral mechanisms that may contribute to HTW during marathon running.

## 3. Glycogen Depletion and Fueling Failure

### 3.1. Glycogen Depletion and Carbohydrate Availability

For many years, glycogen depletion was considered the principal physiological mechanism underlying the development of HTW during marathon running. This concept originated from classical studies demonstrating that endurance exercise capacity is strongly influenced by the availability of endogenous CHO stores, particularly muscle glycogen [9,38]. These investigations showed that athletes who began exercise with higher glycogen stores were able to sustain prolonged exercise for longer durations, establishing glycogen availability as a key determinant of endurance performance.

During marathon running, CHO provide a substantial proportion of the energy required to sustain race pace. Energy is derived from both muscle glycogen and circulating blood glucose, with hepatic glycogen serving an important role in maintaining glucose availability as exercise duration increases [10,39,40]. Reduced CHO availability may also have central consequences. During prolonged exercise, maintenance of blood glucose is important not only for working skeletal muscle but also for cerebral energy availability and the central regulation of effort [39,41]. When hepatic glycogen becomes progressively depleted and exogenous CHO intake is insufficient, reduced blood glucose availability may contribute to central fatigue, increased perceived exertion, impaired concentration, and altered pacing decisions [41]. Gluconeogenic pathways may partly compensate for declining hepatic glycogen availability. In this context, the glucose–alanine cycle may contribute to blood glucose maintenance, as alanine released from working skeletal muscle can be transported to the liver and converted into glucose through gluconeogenesis [42]. However, this compensatory pathway may be insufficient to fully preserve glucose availability when exercise duration, intensity, environmental strain, or inadequate fueling increase metabolic demand. Because endogenous glycogen stores are finite, prolonged exercise progressively reduces CHO availability, particularly when athletes compete at intensities close to their physiological thresholds [8]. When glycogen concentrations decline to critically low levels, the capacity to sustain high rates of CHO oxidation becomes compromised. Consequently, runners become increasingly dependent on fat oxidation to support energy production. Although fat stores are abundant, fat metabolism cannot generate ATP at the same rate as CHO metabolism. This reduction in energy turnover capacity may require a decrease in running speed and has traditionally been proposed as the primary metabolic explanation for the marked slowing frequently observed during the final stages of the marathon [8].

In addition to substrate depletion, lactate kinetics and acid–base balance may contribute to late-race fatigue. Lactate should not be interpreted simply as a metabolic waste product, because it can also serve as an oxidizable substrate and shuttle molecule during exercise [43,44]. In this context, lactate can support energy production by allowing carbon derived from glycolysis to be transported and oxidized in more oxidative muscle fibers, the heart, and other tissues [43,44]. During marathon running, rational utilization of lactate depends less on deliberately increasing lactate accumulation and more on maintaining an appropriate balance between lactate production, clearance, and oxidation. This balance is supported by adequate aerobic conditioning, sufficient CHO availability, appropriate pacing, and avoidance of unsustainable surges that markedly increase glycolytic flux. However, when aggressive pacing, repeated surges, or progressive fatigue increase glycolytic contribution, lactate production and the associated accumulation of hydrogen ions may exceed removal and buffering capacity, contributing to a reduction in muscle pH [36]. As fatigue develops in fatigue-resistant type I fibers, greater recruitment of type II fibers may increase glycolytic contribution and accelerate metabolite accumulation [45]. These acid–base and metabolic disturbances may impair excitation–contraction coupling, calcium handling, and force production, thereby increasing the physiological and perceptual cost of maintaining pace [37]. Therefore, lactate should be viewed as both a useful oxidative substrate and a marker of excessive metabolic strain when production exceeds clearance, whereas acidosis-related mechanisms should be considered additional contributors to the multifactorial development of HTW rather than isolated causes.

The physiological consequences of low glycogen availability extend beyond energy supply. Glycogen depletion has been associated with impaired excitation–contraction coupling, disturbances in calcium handling within skeletal muscle, and reductions in force-generating capacity [46]. Furthermore, low glycogen availability may increase perceived exertion and contribute to the sensation of fatigue, thereby reducing the athlete’s ability to sustain the intended race pace. From a psychophysiological perspective, this is important because endurance performance depends not only on metabolic capacity, but also on the athlete’s ability to tolerate increasing effort and continue regulating pace under progressive physiological strain [5,32,35]. Thus, glycogen depletion may contribute to HTW both directly, by reducing substrate availability and force-generating capacity, and indirectly, by increasing perceived effort and reducing the tolerability of the intended pace. These observations explain why glycogen depletion became the dominant framework for understanding HTW. Consequently, strategies aimed at increasing CHO availability before and during exercise became central components of marathon preparation [10,11]. However, glycogen depletion alone is unlikely to account for the full complexity of late-race performance collapse, particularly given that many athletes continue to experience substantial performance deterioration despite following contemporary evidence-based nutritional strategies [4,10,11]. Therefore, while reduced glycogen availability remains a fundamental component of the traditional HTW model, it should be viewed as one contributor within a broader multifactorial and psychophysiological framework involving thermoregulatory, neuromuscular, gastrointestinal, perceptual, and pacing-related factors.

### 3.2. Fueling Failure During Marathon

Because endogenous glycogen stores are finite, nutritional strategies aimed at maintaining CHO availability during exercise have become a cornerstone of marathon preparation. Contemporary guidelines recommend CHO loading before a competition and regular CHO ingestion throughout the race to preserve glycogen stores, maintain blood glucose concentrations, and support endurance performance [10,11].

The timing of CHO supplementation is also important. During marathon running, CHO intake should generally be initiated early and maintained regularly throughout the race, rather than delayed until substantial fatigue or symptoms of HTW have already developed [10,11,47]. Early and repeated CHO intake may help maintain blood glucose availability, reduce excessive reliance on endogenous glycogen stores, and support sustained exercise intensity. However, the optimal timing and dose should be individualized because gastrointestinal tolerance, intestinal absorption, feeding behavior, and endocrine responses to nutrient ingestion may vary considerably among athletes [14,47]. Although intestinal nutrient sensing and incretin-related responses, including GLP-1, may influence gastrointestinal and glucoregulatory function after CHO ingestion, these responses occur within the broader endocrine and metabolic context of prolonged exercise [48]. Therefore, race-day CHO supplementation should be planned to maintain effective CHO availability while minimizing gastrointestinal burden, rather than delayed in an attempt to preserve endogenous glycogenolysis.

Current recommendations generally support CHO intake rates of 60–90 g·h^−1^ during prolonged endurance exercise when multiple transportable CHOs are used [10,47]. More recently, attention has shifted toward even higher intake rates. Experimental studies have demonstrated that selected well-trained endurance athletes may tolerate CHO intakes approaching 120 g·h^−1^ when appropriate gut-training strategies are implemented, although performance benefits and gastrointestinal tolerance remain highly individualized [12,13]. Furthermore, contemporary elite marathon performances have been associated with aggressive fueling strategies designed to maximize exogenous CHO availability throughout competition [11].

The optimal CHO intake should therefore be determined individually rather than applied as a fixed universal target. A practical approach is to begin with guideline-based ranges according to expected race duration, exercise intensity, athlete experience, and the planned use of multiple transportable CHO, and then refine the target during marathon-specific training [10,11,47]. Long runs and race-pace sessions should be used to test the amount, type, timing, concentration, and delivery format of CHO under conditions that resemble competition [14,47]. The optimal intake for a given runner can be considered the highest CHO dose that supports stable energy availability and pacing without provoking gastrointestinal symptoms or disrupting fluid intake and race execution [13,14,29,47]. Therefore, athletes should monitor CHO intake, gastrointestinal tolerance, perceived effort, hydration behavior, environmental conditions, and pacing responses during training before adopting a race-day fueling plan [10,11].

Despite considerable advances in sports nutrition, fueling failure may still occur among marathon runners. Inadequate pre-race CHO loading, insufficient CHO intake during competition, delayed initiation of fueling, or failure to adhere to a planned nutritional strategy may accelerate glycogen depletion and increase the likelihood of late-race fatigue [8,10]. Recreational runners appear particularly vulnerable, as surveys indicate substantial variability in fueling practices and frequent reliance on subjective judgment rather than evidence-based recommendations [49]. Thus, fueling failure may directly reinforce the traditional glycogen-depletion model by reducing endogenous and exogenous CHO availability during the later stages of the marathon [8,10]. Importantly, fueling failure cannot be explained solely by the quantity of CHO consumed. Surveys of endurance athletes suggest that many runners possess adequate knowledge regarding the importance of CHO intake but often fail to translate this knowledge into effective race-day practices [50]. From a psychophysiological perspective, this knowledge-practice gap may reflect not only nutritional planning, but also behavioral execution under fatigue, gastrointestinal discomfort, perceived effort, and changing race demands. In addition, considerable inter-individual variability exists in gastrointestinal tolerance, CHO absorption, and feeding behavior during prolonged exercise [13,49].

Perhaps most importantly, the persistence of HTW despite increasingly sophisticated nutritional strategies suggests that inadequate CHO availability is not the sole determinant of late-race performance collapse. Some athletes may still experience substantial late-race performance deterioration despite following evidence-based fueling recommendations, whereas others successfully maintain their pace under comparable nutritional conditions [4,16]. These observations indicate that fueling failure should be viewed as one potential contributor to HTW rather than its sole cause. In practice, the effectiveness of fueling depends not only on substrate availability, but also on whether the athlete can maintain intake behavior as physiological strain, perceived effort, and gastrointestinal discomfort increase during the race.

The effectiveness of any fueling strategy ultimately depends not only on the amount of CHO consumed but also on the athlete’s ability to tolerate, absorb, and utilize these nutrients during exercise. Consequently, gastrointestinal function has emerged as an important factor influencing endurance performance and may partially explain why some athletes experience HTW despite apparently adequate nutritional preparation [29,31]. In this context, gastrointestinal dysfunction may reinforce the traditional glycogen-depletion model by limiting the effective availability of exogenous CHO, thereby increasing reliance on finite endogenous glycogen stores during the latter stages of the marathon [8,10,14,29,31]. Gastrointestinal dysfunction may also interfere with fluid and electrolyte replacement, particularly when runners are unable to tolerate CHO-electrolyte solutions or when severe symptoms such as vomiting or diarrhea occur. Under these conditions, electrolyte disturbances may further compound dehydration, cardiovascular strain, perceived effort, and the disruption of fueling behavior. Therefore, fueling failure should be interpreted not only as inadequate CHO intake, but also as a broader failure to maintain effective fluid, electrolyte, and nutrient availability during prolonged exercise [51,52,53].

## 4. Gastrointestinal Dysfunction as a Contributor to the Wall

### 4.1. Exercise-Induced Gastrointestinal Syndrome

Exercise-induced gastrointestinal syndrome (EIGS) refers to a spectrum of gastrointestinal disturbances that may develop during prolonged or strenuous exercise and includes alterations in gastrointestinal integrity, function, and symptom presentation [29,51,52]. EIGS has received increasing attention in endurance sports because gastrointestinal symptoms are frequently reported during marathon, ultra-endurance, and other prolonged endurance events [30,54]. Although gastrointestinal disturbances are not synonymous with HTW, they may impair fluid and CHO intake, compromise nutrient absorption, increase discomfort, and contribute to late-race performance deterioration. In this sense, EIGS may reinforce the traditional glycogen-depletion model by reducing effective exogenous CHO availability and increasing reliance on finite endogenous glycogen stores during the latter stages of the marathon [8,10,14,29,51,52].

The development of EIGS is primarily attributed to interacting circulatory, mechanical, and neuroendocrine mechanisms. During prolonged exercise, blood flow is redistributed away from the gastrointestinal tract toward the working muscles and skin, reducing splanchnic perfusion and potentially compromising intestinal barrier integrity [51,52]. Repetitive mechanical impact during running may further aggravate gastrointestinal symptoms, while exercise-induced stress responses may alter gastrointestinal motility, secretion, and absorption [51,54]. Dehydration may also exacerbate gastrointestinal disturbance by slowing gastric emptying and increasing gastrointestinal discomfort during running [55].

As exercise duration increases, gastrointestinal disturbances may manifest as nausea, abdominal cramping, bloating, reflux, vomiting, urge to defecate, diarrhea, or generalized gastrointestinal discomfort [30,51,52]. The prevalence and severity of these symptoms vary considerably among athletes and appear to be influenced by exercise intensity, nutritional practices, hydration status, environmental conditions, and individual susceptibility [29,30,54]. From a psychophysiological perspective, gastrointestinal symptoms are important because discomfort may increase perceived effort, disrupt attentional focus, and reduce the athlete’s willingness or ability to maintain planned fueling and pacing behaviors during the latter stages of the race [5,32,35].

From a marathon performance perspective, EIGS is particularly relevant because successful race-day fueling depends on the athlete’s ability to ingest, tolerate, absorb, and oxidize CHO and fluids during exercise [10,47,52]. Even when athletes begin competition with optimal glycogen stores and an evidence-based fueling plan, gastrointestinal discomfort may reduce the ability to maintain the planned nutritional strategy, particularly when high CHO intake rates are attempted [13,29,54]. Consequently, EIGS may indirectly contribute to inadequate CHO availability, dehydration, increased perceived exertion, disrupted pacing regulation, and reductions in running speed during the latter stages of the marathon [31,52,55]. This pathway is consistent with the traditional glycogen-depletion model, because impaired CHO intake, absorption, or tolerance may reduce effective CHO availability despite an apparently adequate pre-race nutritional plan [8,10,14,31,52,55].

Importantly, EIGS should not be viewed as an isolated mechanism responsible for HTW. Rather, gastrointestinal dysfunction may interact with other physiological and behavioral stressors, including glycogen depletion, thermoregulatory strain, dehydration, neuromuscular fatigue, perceived exertion, and pacing errors [8,16,21,52]. Within this broader framework, EIGS may increase vulnerability to late-race performance deterioration by limiting the effectiveness of race-day fueling strategies, increasing discomfort, and reducing the athlete’s capacity to maintain physiological homeostasis and behavioral regulation during prolonged exercise [29,31,52]. Thus, gastrointestinal dysfunction may represent an important component of the multifactorial and psychophysiological processes that contribute to the development of HTW.

### 4.2. A Digestive Vulnerability Model

Although gastrointestinal disturbances are common during endurance exercise, substantial inter-individual variability exists in both symptom prevalence and severity. Under similar environmental conditions, exercise intensities, and nutritional practices, some athletes experience little or no gastrointestinal discomfort, whereas others develop symptoms severe enough to impair performance [29,30,55]. This variability suggests that gastrointestinal dysfunction cannot be explained solely by external factors such as CHO intake or environmental heat stress. Rather, susceptibility to exercise-induced gastrointestinal disturbances appears to reflect the interaction between physiological stress and individual vulnerability.

Within this context, digestive vulnerability may be conceptualized as the susceptibility of an athlete to develop gastrointestinal dysfunction when exposed to the combined demands of prolonged endurance exercise. Several factors may contribute to this vulnerability, including a history of gastrointestinal symptoms, exercise intensity and duration, environmental heat stress, dehydration, nutritional practices, and individual differences in gastrointestinal tolerance and absorptive capacity [29,31,52,55]. Aggressive fueling strategies involving high CHO intake rates may further increase gastrointestinal stress in athletes who have not adequately trained the gastrointestinal system to tolerate such nutritional demands [13,47]. From a psychophysiological perspective, digestive vulnerability may also influence how athletes perceive and respond to internal discomfort during competition, thereby affecting feeding behavior, attentional focus, and pacing regulation.

Gut training may reduce digestive vulnerability by improving tolerance to repeated CHO and fluid intake during exercise. Repeated exposure to CHO feeding during training may enhance intestinal CHO transport capacity, potentially through adaptations involving sodium-glucose co-transporter 1 (SGLT1), while also improving gastric comfort and CHO tolerance during prolonged exercise [14,47]. Experimental studies further suggest that gut-training strategies may facilitate the tolerance of high CHO intakes in well-trained athletes [13]. However, gastrointestinal adaptation is likely individualized and may depend on CHO dose, CHO type, feeding frequency, environmental heat stress, hydration status, and prior gastrointestinal symptom history [29,31,52,55]. Therefore, gut training should be implemented progressively during marathon-specific preparation rather than introduced for the first time on race day. Such adaptations may improve the athlete’s ability to maintain planned CHO and fluid intake during competition, thereby reducing one potential pathway leading to late-race performance deterioration [14,29,52].

Digestive vulnerability may influence HTW primarily by disrupting fueling and hydration. Gastrointestinal discomfort may reduce CHO and fluid intake, impair nutrient tolerance, and increase perceived effort during the latter stages of the race [29,30,31,52]. Heat stress and dehydration may further aggravate this process by increasing gastrointestinal disturbance and reducing fluid tolerance [31,55]. Therefore, digestive vulnerability should be viewed as a moderating factor that can increase susceptibility to HTW by compromising the effectiveness of race-day fueling and hydration strategies. In addition, when gastrointestinal discomfort becomes prominent, athletes may consciously or subconsciously reduce pace, alter feeding behavior, or shift attention toward symptom management, further contributing to psychophysiological performance deterioration.

## 5. Thermoregulatory Strain and Environmental Stress

### 5.1. Thermoregulatory Strain During Marathon Running

Thermoregulatory strain represents one of the major physiological challenges encountered during marathon running and may substantially influence endurance performance, particularly during prolonged exercise performed in warm or humid environments [22,53,56]. Because only a small proportion of the energy liberated during muscular contraction is converted into external mechanical work, the majority is released as heat [57,58]. Consequently, marathon runners continuously generate large amounts of metabolic heat that must be dissipated to maintain thermal homeostasis and prevent excessive elevations in body temperature [56,58]. The challenge becomes increasingly pronounced as exercise duration increases, particularly when environmental conditions limit the effectiveness of heat dissipation mechanisms.

To maintain thermal balance, the body relies primarily on sweating and increased skin blood flow. These responses facilitate evaporative and convective heat loss but simultaneously increase cardiovascular demands. As exercise progresses, blood flow is redistributed toward the skin to support heat dissipation while substantial volumes of fluid are lost through sweating [22,56]. This combination may progressively reduce plasma volume and contribute to cardiovascular drift, characterized by gradual increases in heart rate and reductions in stroke volume despite a relatively constant external workload [53]. Consequently, a greater physiological and perceptual effort may be required to sustain a given running speed as race duration increases.

Dehydration further amplifies thermoregulatory stress during marathon running. Fluid losses resulting from prolonged sweating may impair heat dissipation, accelerate the rise in core body temperature, and increase cardiovascular strain [22,53]. Although the relationship between dehydration and performance is complex, evidence suggests that even moderate levels of body mass loss may negatively influence endurance capacity when combined with environmental heat stress [21,53]. Importantly, dehydration may also interact with gastrointestinal dysfunction, potentially reducing fluid tolerance and complicating race-day hydration strategies [31].

As thermal and cardiovascular strain accumulate, athletes frequently adjust pace either consciously or subconsciously in an attempt to limit further physiological stress. Experimental and field-based investigations have demonstrated that elevated core temperature, thermal discomfort, and cardiovascular strain may influence pacing behavior and contribute to reductions in exercise intensity during prolonged endurance events [23,24,56]. From a psychophysiological perspective, thermal strain is particularly relevant because increases in thermal discomfort and perceived exertion may alter the athlete’s tolerance of the intended pace and promote protective reductions in running speed [32,35]. Observations from elite competitions conducted in hot environments further indicate that thermoregulatory responses are closely associated with performance outcomes and the risk of exertional heat illness [21,23].

Therefore, thermoregulatory strain may increase susceptibility to HTW by raising cardiovascular demand, accelerating dehydration, increasing thermal discomfort and perceived exertion, altering pacing behavior, and reducing the capacity to sustain the intended marathon pace [21,23,53,56].

### 5.2. Heat Acclimation, Cooling, and Hydration Strategies

Given the importance of thermoregulatory strain during marathon running, several interventions have been developed to reduce thermal stress and preserve endurance performance. Among these, heat acclimation represents one of the most effective strategies for improving physiological tolerance to exercise in warm environments [56,57]. Repeated exposure to heat induces several adaptations, including plasma volume expansion, earlier onset of sweating, increased sweat rate, improved skin blood flow, and reductions in cardiovascular and thermal strain during exercise [56,57]. These adaptations enhance heat dissipation and may improve endurance performance while reducing the risk of heat-related illness. From a psychophysiological perspective, heat acclimation may also reduce thermal discomfort and perceived exertion during exercise in the heat, thereby improving the athlete’s ability to tolerate and regulate the intended marathon pace.

Cooling interventions have also received considerable attention in endurance sports. Pre-cooling strategies performed before competition, such as cold-water immersion, cooling garments, and ice-slurry ingestion, may lower initial body temperature and increase heat-storage capacity during exercise [57]. Similarly, cooling interventions implemented during competition may attenuate thermal discomfort and reduce perceived exertion, particularly in hot environments [23,57]. By lowering perceptual and thermal strain, cooling strategies may support more stable pacing regulation during periods of elevated environmental stress. The magnitude of benefit likely depends on environmental conditions, exercise duration, and the practicality of implementation during competition.

Hydration remains another fundamental component of thermoregulatory management. Adequate fluid intake may help limit excessive cardiovascular strain and support heat dissipation during prolonged exercise [23,53]. However, hydration strategies should be individualized rather than based on rigid fluid-replacement targets. Excessive fluid consumption may increase the risk of exercise-associated hyponatremia, whereas insufficient fluid intake may exacerbate dehydration, thermal strain, gastrointestinal discomfort, and perceived effort [53,57]. Therefore, hydration planning should consider sweat rate, environmental conditions, race duration, gastrointestinal tolerance, and individual perceptual responses.

## 6. Durability: A New Framework for Understanding Marathon Performance

### 6.1. The Concept and Physiological Underpinnings of Durability

In recent years, durability has emerged as an important concept in endurance physiology because it emphasizes the ability to preserve physiological function during prolonged exercise. Traditionally, endurance athletes have been evaluated using variables measured under rested conditions, including VO_2max_, lactate threshold, and RE. Although these variables remain strong predictors of performance, they do not fully explain why athletes with similar laboratory profiles may display markedly different responses during prolonged endurance events such as the marathon [1,16]. In the marathon context, this variability suggests that durability should be considered as a dynamic property reflecting how well physiological function and performance capacity are maintained as internal load progressively increases [5,32,35].

Maunder et al. [16] formally defined durability as the ability to resist the deterioration of physiological characteristics and performance capacity during prolonged exercise. According to this framework, athletes with superior durability are better able to maintain physiological function, exercise intensity, and performance despite the progressive accumulation of fatigue. Importantly, the physiological phenomena underlying durability were recognized before the term itself became widely used. Earlier investigations by Clark et al. [59,60,61] demonstrated that prolonged exercise can induce progressive increases in oxygen uptake, heart rate, ventilation, and perceived exertion despite maintenance of a constant external workload. These observations showed that physiological and perceptual responses are not static during prolonged exercise but may drift progressively as fatigue accumulates.

Reduced durability may manifest through deterioration in several key determinants of endurance performance. RE may worsen during prolonged running, increasing the oxygen cost required to maintain a given speed [18,19,26]. Similarly, physiological thresholds may shift downward following prolonged exercise, reducing the speed that can be sustained before substantial metabolic disturbance occurs [18]. Recent work by Evans et al. [62] further supports this concept by showing that major determinants of endurance performance assessed under fresh conditions may be altered following prolonged exercise. Together, these findings indicate that endurance capacity should not be viewed as a fixed attribute, but as a dynamic property that may change substantially as exercise duration increases.

Cardiorespiratory responses are also central to durability. During prolonged exercise, heart rate commonly increases despite a stable external workload, a phenomenon often referred to as cardiovascular drift. This response may be accompanied by increased physiological strain, particularly when exercise is performed in warm environments or under conditions of dehydration [24]. As a result, maintaining marathon pace may require progressively greater cardiovascular and perceptual effort as race duration increases. This increasing effort requirement is important because the ability to sustain a given pace depends not only on the preservation of physiological function, but also on whether the athlete can tolerate the rising perceived cost of maintaining that pace [5,35].

From a marathon perspective, durability is particularly relevant because the event requires athletes to sustain a high relative intensity for more than two hours while coping with metabolic, thermoregulatory, neuromuscular, gastrointestinal, perceptual, and pacing-related stressors. Therefore, the ability to preserve physiological function over time may be as important as the absolute magnitude of physiological capacity measured before exercise [1,16]. In this context, HTW may reflect not only depleted energy stores but also a progressive loss of durability across multiple physiological systems, accompanied by increasing perceived effort and impaired regulation of the intended marathon pace.

### 6.2. Durability as a Modifiable Determinant of Marathon Performance

Durability may help explain why some runners maintain pace during the latter stages of the marathon whereas others experience substantial performance collapse. Athletes with superior durability appear better able to preserve RE, physiological thresholds, cardiovascular stability, and exercise intensity despite accumulating fatigue [16,17,18,19,25,26,62]. Conversely, athletes with reduced durability may experience earlier physiological drift, greater energetic cost, and a progressive reduction in the margin between sustainable and unsustainable exercise intensity. In practical terms, a pace that is sustainable early in the marathon may become progressively less tolerable as internal load increases.

Importantly, durability may be at least partly modifiable through training. Recent evidence suggests that higher training volumes and regular exposure to long-duration running are associated with better RE durability in performance-matched runners [19]. This finding is particularly relevant for marathon preparation, because regular long runs may provide a specific stimulus for improving resistance to physiological deterioration during prolonged running. In this sense, durability may represent not only an explanatory construct but also a practical training target. Training interventions that improve durability may help athletes maintain physiological function, movement efficiency, and pace stability for longer before late-race deterioration occurs.

The concept of durability complements traditional determinants of endurance performance. VO_2max_, lactate threshold, and RE measured under rested conditions remain important, but they provide an incomplete picture of marathon performance capacity. The ability to preserve these characteristics under fatigue may determine whether a given marathon pace remains sustainable during the final stages of the race [1,16]. Thus, durability adds a temporal dimension to the classical model of endurance performance by emphasizing how physiological function is maintained over time.

Within the context of HTW, reduced durability may represent the common pathway through which several stressors contribute to the loss of marathon pace. Glycogen depletion, thermoregulatory strain, dehydration, gastrointestinal dysfunction, neuromuscular fatigue, biomechanical deterioration, and pacing errors may each accelerate physiological deterioration during the race [6,7,8,21,27,29,52,53]. When this cumulative burden exceeds the athlete’s capacity to sustain the intended pace, late-race performance collapse may occur [1,4,16]. Accordingly, improving durability may reduce susceptibility to HTW by increasing resistance to the progressive physiological and performance deterioration that develops during the marathon.

## 7. Neuromuscular Fatigue and Biomechanical Deterioration

### 7.1. Neuromuscular Fatigue During Prolonged Running

Neuromuscular fatigue represents an important contributor to performance deterioration during prolonged running. During marathon and ultra-endurance events, repeated muscle contractions over thousands of strides may progressively impair the ability of the neuromuscular system to generate and transmit force effectively [27,28,63,64]. This fatigue may involve peripheral mechanisms, such as impaired excitation–contraction coupling, reduced muscle fiber force production, and muscle damage, as well as central mechanisms, including reduced voluntary activation and altered motor drive [63,65].

At the cellular level, disturbances in muscle ion homeostasis may further contribute to neuromuscular fatigue during prolonged running. Repeated contractions can promote sodium influx and potassium efflux, altering sarcolemmal and transverse-tubular electrochemical gradients, reducing membrane excitability, impairing action-potential propagation, and contributing to excitation–contraction coupling failure [37]. In addition, mechanical loading and excessive reactive oxygen species (ROS) production may perturb sarcolemmal and transverse-tubular membrane function, calcium handling, and force production [37,66]. These peripheral disturbances may contribute to the subjective sensation of “heavy legs,” reduced force-generating capacity, and an increased energetic cost of maintaining pace. ROS-related oxidative stress may also contribute to central or neuronal fatigue by affecting neuronal excitability, neurotransmission, afferent feedback, and motor drive, thereby increasing perceived effort and reducing the capacity to sustain the intended pace [67]. Therefore, ionic disturbances and ROS-related mechanisms should be considered additional contributors to the multifactorial onset of HTW, particularly when metabolic, thermal, neuromuscular, and perceptual strain accumulate during the final stages of the marathon.

Prolonged running has been shown to induce reductions in maximal voluntary force, explosive strength, and stretch-shortening cycle function [63,64,68]. These changes may compromise running speed, particularly during the latter stages of a marathon when metabolic, thermal, and perceptual strain are already elevated. Although many studies assess neuromuscular function after race completion, the processes leading to reduced force production and altered muscle function likely develop progressively during the race itself [27,28,63].

The effect of neuromuscular fatigue on marathon performance is likely mediated by several pathways. As force-generating capacity declines, athletes may need to recruit additional motor units or increase relative effort to maintain the same running speed [63,65]. This can increase perceived exertion and accelerate fatigue. In addition, reductions in muscle stiffness, tendon function, and elastic energy return may impair running mechanics and increase the energetic cost of running, linking neuromuscular fatigue with deterioration in RE [25,26,64,65]. As a result, the intended marathon pace may become progressively more costly from both energetic and perceptual perspectives.

Accordingly, neuromuscular fatigue should be viewed as more than a post-race outcome. As fatigue accumulates, reduced force production and altered muscle function may contribute to the inability to sustain target pace, particularly when combined with glycogen depletion, thermoregulatory strain, dehydration, biomechanical deterioration, and reduced durability [8,16,21,53]. Within the broader HTW framework, neuromuscular fatigue may therefore reduce the mechanical reserve required to maintain pace when other physiological stressors are already accumulating.

### 7.2. Biomechanical Deterioration and Running Economy

Prolonged running may also induce biomechanical alterations that contribute to deterioration in RE. As fatigue accumulates, runners may exhibit changes in stride length, stride frequency, ground contact time, vertical oscillation, leg stiffness, and joint kinematics [26,64,69]. These adjustments may initially represent compensatory strategies to maintain speed, but they may also increase the energetic cost of running when neuromuscular fatigue becomes substantial.

A key mechanism linking biomechanical deterioration with performance decline is impairment of the stretch-shortening cycle. During distance running, efficient storage and return of elastic energy in the muscle-tendon unit helps reduce the metabolic cost of movement. However, prolonged running may reduce muscle-tendon stiffness, impair reactive strength, and decrease elastic energy return [28,64,68]. These changes may increase the muscular work required to sustain a given pace and contribute to worsening RE. Fatigue-related biomechanical changes may also alter muscle recruitment patterns and increase mechanical loading on already fatigued tissues. As force production becomes less efficient, runners may adopt less economical movement patterns that increase oxygen cost and perceived effort [25,26]. This may be particularly important when other stressors have already reduced the physiological reserve available to compensate for the rising cost of running.

Collectively, neuromuscular fatigue and biomechanical deterioration may increase the energetic cost of running, reduce mechanical efficiency, and amplify perceived effort. These changes provide a plausible pathway through which prolonged running stress may contribute to difficulty maintaining marathon pace during the latter stages of the race. Within the broader HTW framework, biomechanical deterioration may therefore accelerate the transition from a sustainable pace to one that becomes increasingly costly and difficult to regulate.

## 8. Pacing and Development of the Marathon Wall

### 8.1. Pacing Strategies in Marathon Running

Pacing strategy is widely recognized as one of the major determinants of endurance performance because it regulates the distribution of effort throughout exercise and influences the rate at which physiological stress accumulates [6,7]. In marathon running, pacing reflects the continuous interaction between metabolic demands, thermoregulatory constraints, neuromuscular fatigue, and perceptual responses, allowing athletes to manage available physiological resources over prolonged durations [1,6]. From this perspective, pacing can be understood as a psychophysiological regulation strategy, in which athletes continuously adjust speed based on internal sensory feedback, perceived effort, prior experience, expected remaining distance, and environmental demands [32,35].

Three pacing patterns are commonly described in marathon running: positive split, even pacing, and negative split. Positive split pacing is characterized by a faster first half followed by progressive slowing during the second half of the race, whereas even pacing aims to maintain relatively constant speed throughout competition [4,70]. In contrast, negative split pacing involves completing the second half of the race faster than the first half and has been associated with superior performance and more favorable physiological and perceptual responses [3,71]. Observational studies have shown that substantial late-race reductions in speed are common among recreational marathon runners and may reflect pacing errors, fatigue accumulation, or both [4]. In contrast, elite athletes often exhibit smaller pacing fluctuations and demonstrate a greater ability to maintain speed during the latter stages of competition [70,72]. These differences may reflect superior physiological characteristics, greater durability, enhanced perceptual regulation, and more effective race execution strategies [1,16].

Recent work has emphasized that pacing should not be viewed solely as a tactical decision but rather as a dynamic regulatory process involving continuous adjustments in response to internal and external stressors [6,71]. Accordingly, successful pacing requires the integration of physiological capacity, experience, environmental conditions, psychological factors, and moment-to-moment perceptions of effort. Because pacing determines the rate at which metabolic, thermoregulatory, and neuromuscular strain accumulate, it may play a central role in determining whether athletes maintain performance or experience substantial late-race deterioration.

Mental resilience may also influence how runners respond to increasing fatigue, discomfort, and pain during the latter stages of the marathon. In this context, mental resilience should not be interpreted as simply ignoring pain or overriding protective signals, but rather as the capacity to tolerate rising perceived effort, maintain attentional control, regulate negative affect, and continue making appropriate pacing and fueling decisions under progressive physiological strain [5,35,73]. This may be particularly relevant to HTW because late-race slowing often reflects not only reduced physiological capacity, but also changes in effort perception, motivation, attentional focus, and the willingness or ability to sustain the intended pace [33,34,35]. Therefore, psychological resilience and fatigue tolerance should be considered additional modulators of HTW susceptibility, interacting with metabolic, thermoregulatory, gastrointestinal, neuromuscular, and pacing-related factors.

### 8.2. Pacing Errors and Late-Race Performance Collapse

Pacing errors represent one of the most important behavioral contributors to late-race performance collapse in marathon running. An excessively fast early pace may increase metabolic cost, accelerate glycogen utilization, elevate lactate accumulation, and increase perceived exertion before the athlete has reached the most demanding stages of the race [4,6,7,8]. Although a fast start may initially feel manageable, particularly in well-trained runners, it may progressively reduce the physiological reserve required to sustain pace during the final 10–12 km.

Aggressive early pacing may also amplify thermoregulatory and cardiovascular strain. Higher running speeds increase metabolic heat production and cardiovascular demand, which may accelerate dehydration, cardiovascular drift, and increases in core temperature, especially in warm or humid conditions [21,22,23,24,53]. Consequently, the physiological cost of maintaining pace may rise disproportionately during the latter stages of the marathon, increasing the likelihood of involuntary slowing. Large-scale analyses of marathon pacing have shown that substantial late-race slowing is common among recreational runners and is frequently associated with performance loss during the final stages of the race [4]. In many cases, this pattern may reflect a mismatch between the selected early pace and the athlete’s actual capacity to sustain that intensity over the full marathon distance. Thus, HTW may occur not simply because the athlete lacks sufficient physiological capacity, but because early pacing decisions accelerate the depletion of metabolic, thermal, neuromuscular, and perceptual reserves.

Pacing errors may also interact with gastrointestinal function and fueling behavior. Starting too fast may increase sympathetic activation, reduce gastrointestinal perfusion, and impair tolerance of CHO and fluid intake during the race [29,31,52]. This may lead runners to delay or reduce fueling, thereby increasing vulnerability to inadequate CHO availability and further late-race deterioration [8,29,52]. In this way, pacing can act as an upstream regulator that influences several other mechanisms involved in HTW [6,7]. Therefore, pacing should not be viewed merely as a tactical choice, but as a psychophysiological regulation strategy. A poorly regulated pace may accelerate the development of glycogen depletion, thermoregulatory strain, gastrointestinal dysfunction, neuromuscular fatigue, biomechanical deterioration, perceived exertion, and reduced durability [4,6,7,8,16,21,52]. Conversely, more conservative or well-distributed pacing may reduce the rate at which these stressors accumulate and lower susceptibility to late-race collapse [4,6,7,70,71].

## 9. An Integrative Model of the Marathon Wall

**Figure 1:**
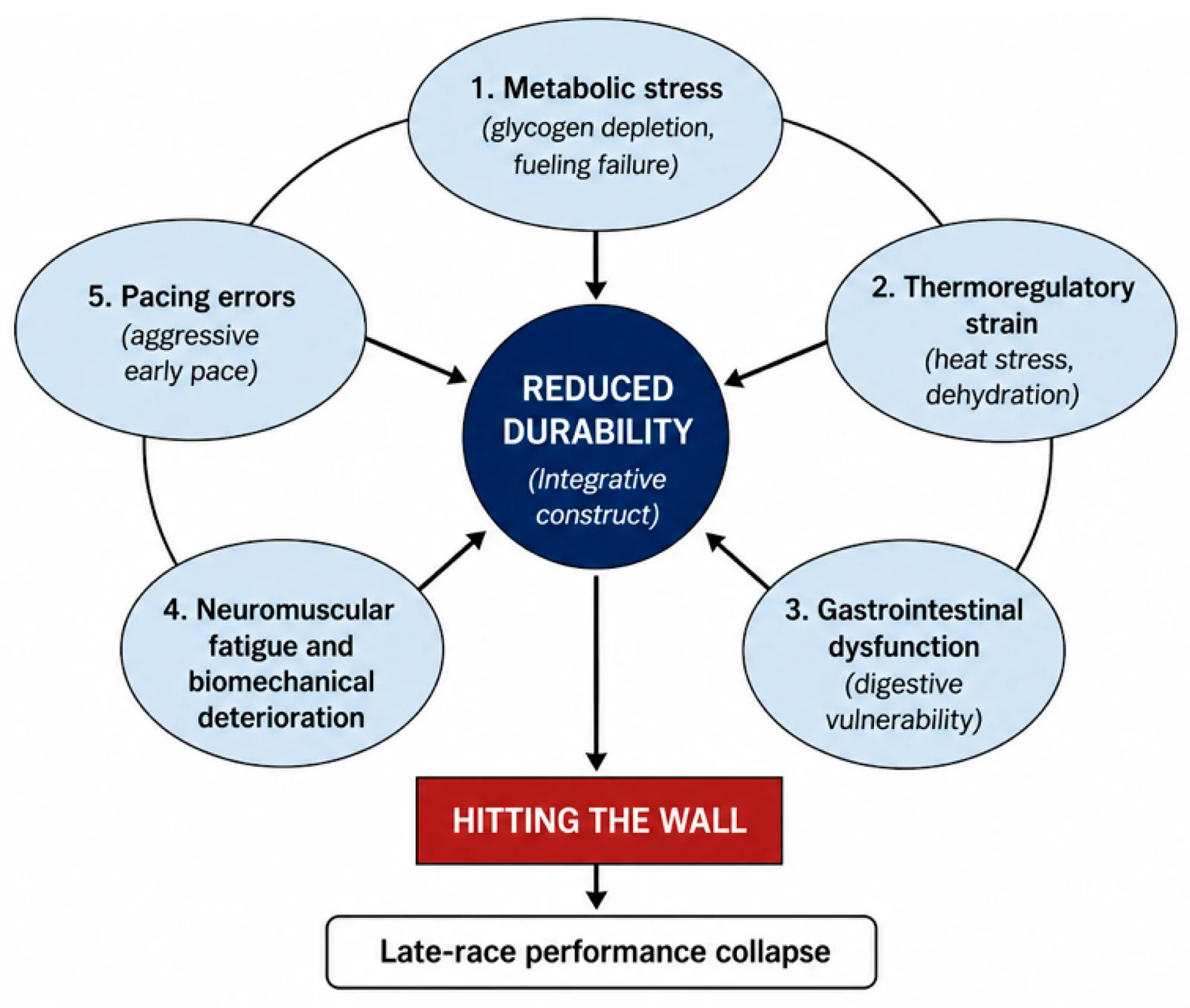
Integrative psychophysiological and durability-based model of hitting the wall in marathon running. Metabolic stress, thermoregulatory strain, gastrointestinal dysfunction, neuromuscular fatigue and biomechanical deterioration, and pacing errors may interact to reduce durability. Within this framework, reduced durability is conceptualized as an integrative construct that links the cumulative effects of these physiological, perceptual, and behavioral stressors to hitting the wall and late-race performance collapse.

Traditional explanations of HTW have focused primarily on glycogen depletion and reduced CHO availability [8,9,10,38]. Although this model remains physiologically important, it does not fully explain why late-race collapse persists despite contemporary fueling strategies or why runners exposed to similar race demands may display markedly different outcomes [1,4,16]. HTW is therefore better understood as the result of interacting metabolic, thermoregulatory, gastrointestinal, neuromuscular, hydration-related, biomechanical, perceptual, and pacing-related stressors that progressively reduce the athlete’s capacity to sustain the intended race pace (Table 1). Within this framework, reduced durability represents the central construct linking these stressors to the development of HTW (Figure 1). Reduced durability should therefore be interpreted not as an isolated mechanism, but as the integrative expression of progressive physiological and psychophysiological deterioration across interacting systems.

**Figure 2:**
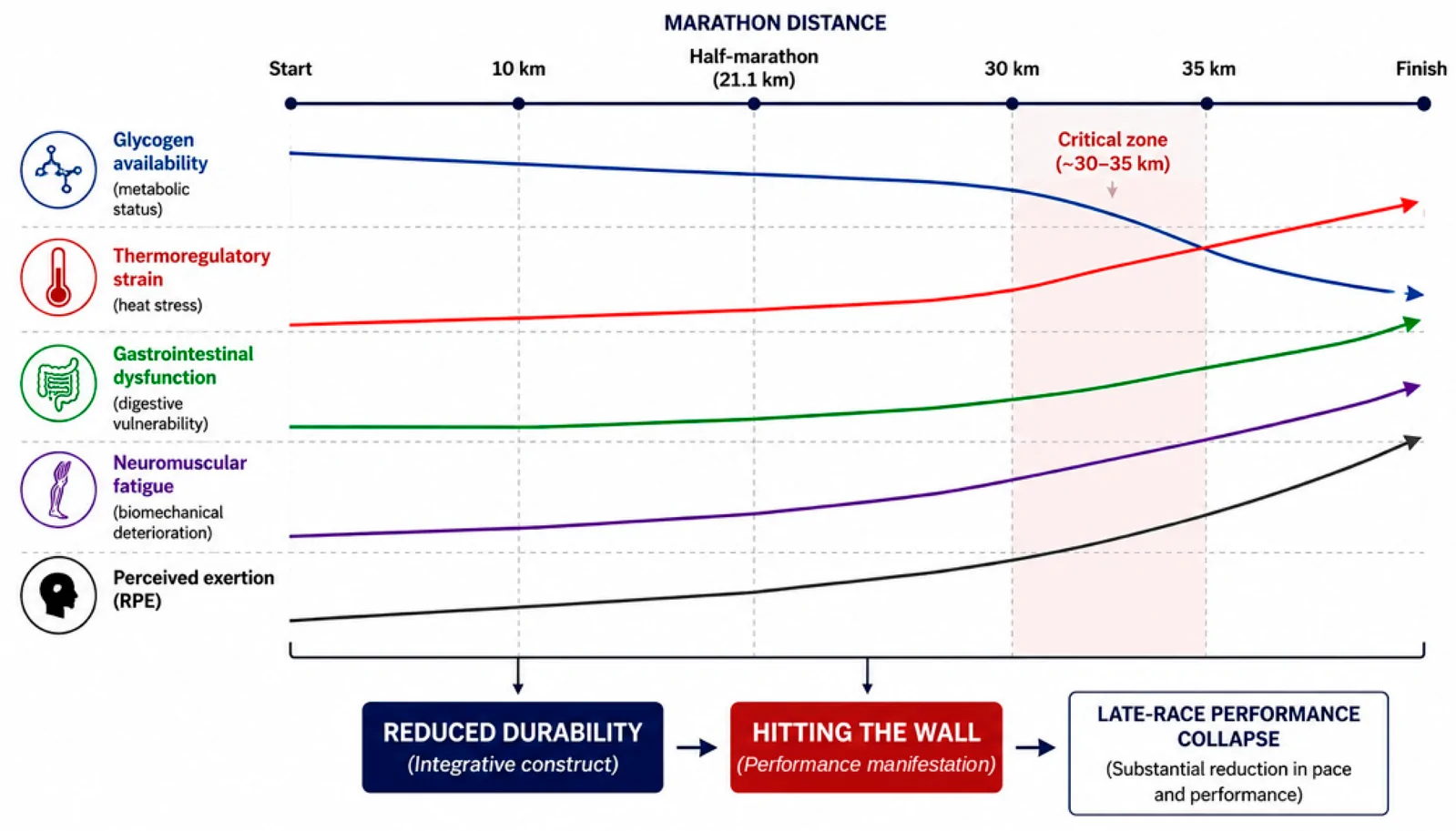
Progressive development of physiological and psychophysiological strain across the marathon distance. Glycogen availability may progressively decline, whereas thermoregulatory strain, gastrointestinal dysfunction, neuromuscular fatigue, and perceived exertion may increase as the race progresses. The 30–35 km segment is presented as a critical zone in which interacting physiological, perceptual, and behavioral stressors may exceed the athlete’s durability and increase susceptibility to hitting the wall and late-race performance collapse.

These stressors do not operate independently. Glycogen depletion may impair force production and increase perceived exertion [46], while dehydration and thermoregulatory strain may accelerate cardiovascular drift, gastrointestinal disturbance, thermal discomfort, and perceptual strain [21,24,31,53]. Gastrointestinal dysfunction may compromise CHO and fluid intake [29,52], neuromuscular fatigue and biomechanical deterioration may increase the energetic and perceptual cost of running [25,26,27,28], and pacing errors may accelerate the accumulation of stress across several systems [6,7]. Thus, the likelihood of HTW depends not only on the severity of each stressor, but also on how these stressors interact over time and influence the athlete’s ability to regulate effort and pace (Figure 2).

Durability provides the conceptual link between these mechanisms and marathon performance. Athletes with greater durability appear better able to preserve physiological thresholds, RE, cardiovascular stability, gastrointestinal tolerance, neuromuscular function, and pace stability as fatigue accumulates [16,17,18,19,25,26,62]. In contrast, reduced durability may cause a previously sustainable pace to become progressively unsustainable, especially when combined with heat stress, fueling disruption, dehydration, gastrointestinal symptoms, neuromuscular fatigue, biomechanical deterioration, or aggressive pacing [1,16,59,61]. Accordingly, HTW may be conceptualized as a failure of durability across interacting physiological and psychophysiological systems rather than the isolated consequence of glycogen depletion.

This model shifts the interpretation of HTW from “running out of fuel” toward “losing the capacity to sustain the chosen pace.” CHO availability remains important, but it is only one component of a broader regulatory system. From this perspective, late-race collapse occurs when the cumulative physiological burden and perceived cost of maintaining pace exceed the athlete’s remaining capacity for durable performance. Prevention strategies should therefore target not only fueling, but also thermal regulation, gastrointestinal tolerance, neuromuscular resilience, pacing control, hydration management, perceptual regulation, and durability development [7,10,19,21,52] (Table 2).

## 10. Practical Strategies to Reduce Susceptibility to the Marathon Wall

### 10.1. Training Interventions

Training strategies aimed at reducing susceptibility to HTW should target the systems most likely to fail during the latter stages of the marathon. Regular long runs and higher training volumes may improve RE durability and enhance the ability to resist physiological deterioration during prolonged running [19]. These sessions may also help athletes become more familiar with the progressive increase in internal load that occurs during marathon-specific exercise, thereby supporting more effective pace regulation under fatigue. Resistance training may further support marathon performance by improving RE, force production, and fatigue resistance without compromising aerobic adaptations [74,75,76]. These adaptations may help preserve neuromuscular function, movement efficiency, and mechanical reserve when fatigue accumulates.

Heat acclimation is particularly relevant when competition is expected in warm environments. Repeated heat exposure can improve thermoregulatory responses, expand plasma volume, reduce cardiovascular strain, and enhance exercise tolerance [56,77]. By reducing thermal strain and discomfort, heat acclimation may also support the athlete’s ability to maintain effort and pace in warm conditions. Gut training may also be useful because repeated CHO feeding during exercise can improve gastrointestinal tolerance and the ability to maintain fueling during competition [13,29,52]. This may reduce the likelihood that gastrointestinal discomfort disrupts fueling behavior or contributes to late-race pacing deterioration. Together, these interventions target durability across metabolic, thermal, gastrointestinal, neuromuscular, and perceptual domains (Table 2).

### 10.2. Race-Day Strategies

Race-day strategies should translate training adaptations into effective execution. CHO loading and CHO ingestion during exercise remain central for maintaining substrate availability and reducing susceptibility to late-race fatigue [10,11]. Current guidelines generally support 60–90 g·h^−1^ during prolonged endurance exercise when multiple transportable CHO are used [10,47]. Higher intakes approaching 120 g·h^−1^ may be tolerated by selected well-trained athletes after appropriate gut training, but this should not be interpreted as a universal recommendation [11,12,13]. For many recreational runners, attempting very high CHO intakes without prior practice may increase gastrointestinal distress, compromise fueling, and disrupt pacing execution.

Legal ergogenic aids may also be considered within an individualized race-day plan. Caffeine is one of the most widely used and evidence-supported ergogenic aids in endurance sport and may reduce susceptibility to late-race slowing through both central and peripheral mechanisms [78,79]. From a cerebral and neuronal perspective, caffeine may increase alertness, reduce perceived exertion, and modulate central fatigue, thereby helping runners maintain attentional focus and tolerate rising effort during the latter stages of the marathon [78]. From a metabolic perspective, caffeine may also support endurance performance, although its practical effects depend on dose, timing, habituation, gastrointestinal tolerance, and individual responsiveness [78]. Recent meta-analytic evidence in endurance running indicates that caffeine can improve time-to-exhaustion performance and may produce small benefits in running time-trial performance [79]. Therefore, caffeine should be tested during marathon-specific training rather than introduced for the first time on race day, particularly because excessive or unfamiliar intake may increase gastrointestinal discomfort, anxiety, or pacing disruption [78].

Hydration and cooling strategies should be individualized according to sweat rate, environmental conditions, race duration, gastrointestinal tolerance, and perceptual responses [23,53,80]. Adequate hydration may help limit excessive cardiovascular and thermal strain, whereas overdrinking may increase the risk of exercise-associated hyponatremia. Cooling strategies may also help reduce thermal discomfort and perceived effort, particularly in warm environments.

Electrolyte replacement should also be individualized, particularly during prolonged races performed in warm or humid conditions. Sodium losses vary widely among runners according to sweat rate, sweat sodium concentration, exercise duration, environmental conditions, and fluid intake behavior [58,81]. Gastrointestinal dysfunction may further complicate electrolyte replacement by reducing tolerance to CHO-electrolyte solutions, impairing fluid and nutrient absorption, or, in more severe cases, increasing fluid and electrolyte losses through vomiting or diarrhea [29,51,52]. Therefore, electrolyte compensation should be based on an individualized hydration plan that is practiced during training and adjusted according to sweat rate, expected race duration, environmental conditions, gastrointestinal tolerance, and signs of excessive fluid or sodium loss. Sodium-containing fluids, sports drinks, or oral rehydration-type solutions may be useful when tolerated, but aggressive fluid intake should be avoided because overdrinking can increase the risk of exercise-associated hyponatremia [58,81].

Pacing should be planned according to the athlete’s training history, realistic race capacity, course profile, and environmental conditions. Conservative or evenly distributed pacing may reduce early metabolic and thermal stress, preserve physiological reserve, and support more stable effort regulation. In contrast, aggressive early pacing can accelerate physiological and perceptual strain and increase susceptibility to HTW [4,6,7,15,70].

Race-day strategies should also be adjusted according to environmental conditions. In cool conditions, thermoregulatory strain is generally lower, and susceptibility to HTW may depend more strongly on CHO availability, pacing errors, neuromuscular fatigue, and the ability to tolerate prolonged effort. In temperate, warm, or humid conditions, however, heat storage, dehydration, cardiovascular drift, thermal discomfort, and gastrointestinal symptoms may develop earlier and interact with substrate depletion and pacing regulation [82,83]. Therefore, marathon performance collapse in hot environments should not be interpreted only as a metabolic failure, but as an interaction between metabolic, thermoregulatory, cardiovascular, gastrointestinal, and perceptual stressors [82,83]. Accordingly, heat acclimation, hydration, cooling, CHO intake, and pacing should be adjusted to the expected environmental strain [82,83,84].

### 10.3. Individualization and Monitoring

Because runners differ in durability, sweat rate, gastrointestinal tolerance, heat responsiveness, pacing behavior, perceptual responses, and training history, prevention strategies should not be applied uniformly [1,16,47,53]. Individualized race planning should integrate previous long-run responses, fueling tolerance, gastrointestinal symptoms, environmental conditions, perceived effort, and realistic pacing targets.

Monitoring can support this process by tracking internal and external responses to training and competition stress. Measures such as heart rate, perceived exertion, subjective recovery, pace variability, training load, environmental heat stress, and gastrointestinal symptoms may help identify excessive strain, inadequate readiness, or poor tolerance of marathon-specific demands [85,86,87]. However, these data should be interpreted alongside athlete feedback and coaching judgment rather than used in isolation. In this context, monitoring is most useful when it helps identify mismatches between the intended race plan and the athlete’s physiological and perceptual responses.

This approach shifts prevention from generic marathon advice toward targeted strategies that address the mechanisms most likely to limit performance in each athlete (Table 2). By integrating physiological markers, perceptual feedback, training history, and race-specific responses, individualized monitoring may help reduce susceptibility to HTW and support more effective marathon preparation and race-day decision-making.

## 11. Future Directions

Future research should move beyond isolated explanations of HTW and investigate marathon performance as a dynamic, multisystem and psychophysiological phenomenon. Although glycogen depletion, thermoregulatory strain, gastrointestinal dysfunction, neuromuscular fatigue, biomechanical deterioration, pacing errors, perceptual responses, and reduced durability have often been studied separately, their interaction during real-world marathon competition remains insufficiently understood. Consequently, future studies should focus on how these mechanisms evolve simultaneously throughout the race and how their combined effects contribute to late-race performance collapse [1,4,16].

Advances in wearable technology provide new opportunities for monitoring physiological and perceptual responses during training and competition. Continuous or near-continuous assessment of heart rate, pace variability, environmental heat stress, heart-rate variability, estimated core temperature, perceived exertion, and subjective fatigue may allow earlier detection of excessive physiological and psychophysiological strain before substantial performance deterioration occurs [85,88,89]. However, further research is needed to establish the validity, reliability, and practical application of wearable-derived metrics under competitive marathon conditions.

Artificial intelligence and machine learning approaches may further enhance the prediction and prevention of HTW. By integrating information derived from training history, pacing behavior, environmental conditions, fueling practices, heart-rate responses, gastrointestinal symptoms, perceived exertion, subjective fatigue, and durability-related variables, predictive models may help identify athletes at increased risk of late-race performance collapse [90,91]. Such approaches may facilitate individualized race planning, real-time decision-making, and precision-based interventions designed to preserve performance during prolonged exercise. More broadly, the integration of artificial intelligence with wearable technologies may contribute to the development of digital twins and personalized endurance medicine [92].

Another important area for future investigation concerns the assessment of durability. Although durability is increasingly recognized as an important determinant of endurance performance, standardized approaches for evaluating durability in runners remain limited [1,16]. Recent studies have demonstrated that physiological decoupling, prolonged exercise protocols, and changes in RE may provide practical approaches for assessing durability in both laboratory and field settings [17,26]. Nevertheless, future durability assessments should also consider perceptual and behavioral responses, including perceived exertion, pace stability, and the athlete’s ability to maintain effort regulation under prolonged internal load.

Finally, intervention studies are needed to determine whether targeted strategies can reduce susceptibility to HTW. Future research should examine the combined effects of marathon-specific long runs, strength training, heat acclimation, gut training, individualized fueling, hydration strategies, cooling interventions, perceptual-regulation strategies, and pacing education [7,10,13,19,52,56,74,75,76,77,80]. Such studies may clarify whether improving durability across multiple physiological and psychophysiological systems can enhance resistance to fatigue and reduce the likelihood of substantial late-race performance collapse [1,16].

## 12. Conclusions

Glycogen depletion and reduced CHO availability remain important contributors to marathon fatigue, but they do not fully explain the complexity of HTW [8,10]. Rather than representing an isolated metabolic event, HTW is better understood as the manifestation of reduced durability arising from the cumulative interaction of metabolic, thermoregulatory, gastrointestinal, hydration-related, neuromuscular, biomechanical, perceptual, and pacing-related stressors during marathon running [1,4,16]. This integrative perspective shifts the focus from individual mechanisms toward the athlete’s ability to preserve physiological function, regulate effort, and sustain the intended race pace over time [1,16].

Accordingly, improving durability through marathon-specific training, optimized fueling, gastrointestinal tolerance, thermoregulatory preparation, neuromuscular resilience, pacing regulation, perceptual awareness, and individualized monitoring may reduce susceptibility to HTW and enhance marathon performance [7,10,19,52,56,74,87]. Future studies should determine whether interventions specifically designed to improve durability across physiological and psychophysiological domains can reduce the incidence and severity of HTW in marathon runners. Such approaches may support more individualized marathon preparation and improve race-day decision-making.

## Figures and Tables

**Table 1:** Major mechanisms contributing to HTW.

Mechanism	Principal Physiological Consequences	Potential Contribution to HTW
Glycogen depletion and fueling failure	Reduced CHO availability, increased reliance on fat oxidation	Reduced capacity to sustain marathon pace
Gastrointestinal dysfunction	Impaired CHO and fluid tolerance, reduced nutrient absorption	Disruption of fueling and hydration strategies
Thermoregulatory strain	Hyperthermia, dehydration, cardiovascular drift	Increased physiological cost and pace reduction
Neuromuscular fatigue and biomechanical deterioration	Reduced force production, impaired stretch-shortening cycle, deterioration in RE	Reduced movement efficiency and increased energetic cost
Pacing errors	Excessive early physiological stress and accelerated resource depletion	Earlier onset of fatigue and late-race slowing

HTW: hitting the wall; CHO: carbohydrate; RE: running economy. Note: Reduced durability should be interpreted as the integrative outcome of interacting metabolic, thermoregulatory, gastrointestinal, neuromuscular, biomechanical, perceptual, and pacing-related stressors, rather than as a separate mechanism.

**Table 2:** Practical strategies to reduce susceptibility to HTW during marathon running.

Target Mechanism	Practical Strategy	Physiological and Psychophysiological Rationale	Practical Application
CHO availability	CHO loading and planned CHO intake during the race	Preserves CHO availability, supports blood glucose maintenance, and delays excessive reliance on fat oxidation	Practice race fueling in long runs; individualize intake according to tolerance, with 60–90 g·h^−1^ commonly recommended and up to ~120 g·h^−1^ only in selected gut-trained athletes
Legal ergogenic aids	Individualized caffeine use	May reduce perceived exertion, increase alertness and attentional focus, and support endurance performance through central and peripheral mechanisms	Practice dose and timing during marathon-specific training; avoid unfamiliar or excessive intake on race day, especially in runners prone to gastrointestinal discomfort, anxiety, or sleep-related disruption
Gastrointestinal tolerance	Gut training and repeated practice of race-day nutrition	Improves tolerance to CHO and fluid intake and reduces the risk of fueling disruption	Use planned gels/drinks during long runs and marathon-specific sessions; avoid untested products on race day
Thermoregulatory strain	Heat acclimation and cooling strategies	Reduces cardiovascular and thermal strain, improves heat dissipation, and may lower thermal discomfort and perceived effort	Use heat-acclimation blocks before hot races; consider pre-cooling or per-cooling strategies when appropriate
Hydration-related stress	Individualized hydration planning	Limits excessive dehydration while reducing the risk of overdrinking and exercise-associated hyponatremia	Estimate sweat rate in training; adjust fluid intake according to environmental conditions, race duration, gastrointestinal tolerance, and individual needs
Neuromuscular resilience	Resistance training and marathon-specific muscular endurance work	Improves force production, RE, and resistance to neuromuscular fatigue	Include progressive strength training and hill or marathon-specific sessions without compromising endurance training quality
Durability	Regular long runs and progressive marathon-specific training volume	Enhances the ability to preserve RE, physiological thresholds, pace stability, and overall performance capacity during prolonged exercise	Develop long-run tolerance progressively; include sessions that simulate the metabolic, neuromuscular, and perceptual demands of the final marathon stages
Pacing regulation	Conservative start, even pacing, or negative split strategies	Reduces early resource depletion and slows the accumulation of metabolic, thermal, neuromuscular, and perceptual strain	Set realistic target pace based on current fitness, course profile, and environmental conditions; avoid excessive early surges
Monitoring and individualization	Use of heart rate, RPE, pace variability, environmental conditions, gastrointestinal symptoms, and athlete feedback	Helps identify excessive physiological strain, poor recovery, accelerated drift, or poor tolerance of marathon-specific demands before performance collapse occurs	Interpret data within the context of training history, race goals, environmental stress, and subjective responses rather than relying on single metrics

HTW: hitting the wall; CHO: carbohydrate; RE: running economy; RPE: rating of perceived exertion.

## Data Availability

No new data were created or analyzed in this study. Data sharing is not applicable to this article.
